# Development and Application of a *Rep*‐Targeted SYBR Green I qPCR Assay for Porcine Circovirus 4: Etiological Investigation in China (2022–2025) and Molecular Evolutionary Analysis

**DOI:** 10.1155/tbed/4853360

**Published:** 2026-11-06

**Authors:** Yi Lu, Yanhong Chen, Tong Xu, Yang Zeng, Ling Dong, Zhijing Mei, Xuexiang Yu, Suphot Wattanaphansak, Wentao Li, Qigai He

**Affiliations:** ^1^ National Key Laboratory of Agricultural Microbiology College of Veterinary Medicine Huazhong Agricultural University Wuhan 430070 China hzau.edu.cn; ^2^ The Cooperative Innovation Center for Sustainable Pig Production Huazhong Agricultural University Wuhan 430070 China hzau.edu.cn; ^3^ Department of Veterinary Medicine Faculty of Veterinary Science Chulalongkorn University Henri Dunant Road, Pathumwan Bangkok 10330 Thailand chula.ac.th; ^4^ Wuhan (China-Thailand) Belt and Road Joint Laboratory for Key Technologies in Livestock and Poultry Disease Prevention and Control Wuhan 430070 China

**Keywords:** molecular epidemiology, porcine circovirus type 4, selection pressure, structural modeling, SYBR Green I real-time quantitative PCR

## Abstract

Porcine circovirus (PCV) type 4 (PCV4) is an emerging *Circovirus* whose epidemiological and evolutionary dynamics remain incompletely characterized. We developed a replication‐associated protein (*Rep*)‐targeted SYBR Green I real‐time quantitative PCR assay that showed analytical specificity against tested common porcine pathogens and an estimated 95% limit of detection of 8.22 copies/µL. Among the 1920 diagnostic samples collected from 29 Chinese provinces between 2022 and 2025, 96 tested positive for PCV4 (5.00%; 95% confidence interval [CI]: 4.11%–6.07%). The highest observed positivity was in sows, although rates did not differ significantly among production stages. The capsid (*Cap*) gene‐based phylogenetic analysis indicated that most analyzed sequences belonged to PCV4a. Exploratory Bayesian analyses estimated a global time to the most recent common ancestor around 1996.6 and an evolutionary rate of 4.76 × 10^−4^ substitutions/site/year. Discrete phylogeographic reconstruction identified model‐inferred migration links rather than verified transmission routes. Three candidate positively selected sites (Ser27, Ser53, and Val98) overlapped predicted structural or epitope‐associated regions. Overall, the assay provides a practical molecular tool for PCV4 surveillance, and the epidemiological and evolutionary findings generate hypotheses for future systematic sampling, recombination‐aware genomic analysis, and investigation of possible vertical transmission.

## 1. Introduction

Porcine circoviruses (PCVs), members of the genus *Circovirus* within the family Circoviridae, are among the smallest autonomously replicating animal viruses known to infect animals [1]. The virion is characterized by a nonenveloped icosahedral structure (~13–25 nm in diameter), encompassing a circular single‐stranded DNA genome of 1.7–2.0 kb [2]. According to the taxonomy by the International Committee on Taxonomy of Viruses (ICTV), four distinct species have been confirmed [3]: PCV1 [4], PCV2 [5], PCV3 [6], and PCV type 4 (PCV4) [7]. More recently, a porcine‐associated eukaryotic circular replication‐associated protein (*Rep*)‐encoding single‐stranded DNA (CRESS DNA) virus, tentatively named PCV5, was reported to have a comparatively large genome of 2904nt. However, despite its circular genome organization, phylogenetic analyses suggest that this virus is most closely related to fur seal feces‐associated circular DNA virus (FSfaCV) and clusters outside the family Circoviridae [8].

PCV4 is genetically distinct from other PCVs. Genomic analysis shows that PCV4 shares relatively low nucleotide identity with PCV1 (50.3%), PCV2 (51.5%), and PCV3 (43.2%) [8], while it displays a higher phylogenetic similarity (66.9%) to mink circovirus (MiCV) [7]. Retrospective epidemiological studies indicate that PCV4 may have circulated cryptically in wild swine populations since 2011, with robust evidence of its presence in European wildlife tracing back to the late 1990s and early 2000s [9], providing an extended timeline for understanding its epidemiological evolution. Since its first discovery in China, PCV4 has also been identified in some Asian countries, including South Korea [10], Thailand [11], and Malaysia [12], underscoring the need for continued regional surveillance. Notably, the recent detection of PCV4 in domestic swine in the United States [13] and wild boar populations in Europe [9] suggests that the recognized geographic distribution of PCV4 extends beyond Asia.

Given the genetic distinctiveness and expanding recognized distribution of PCV4, sensitive and specific molecular assays are essential for surveillance. The capsid (*Cap*) gene encodes the major structural protein and contains important antigenic determinants; consequently, it may experience stronger immune‐mediated selection and greater sequence variability than more functionally constrained genomic regions. Sequence variation within primer‐ or probe‐binding sites in the *Cap* gene may impair oligonucleotide binding, reduce amplification efficiency, and increase the risk of false‐negative results. In contrast, the *Rep* gene is generally more conserved and is expected to be constrained by purifying selection [14]. We therefore selected a conserved region of the *Rep* gene as the qPCR target to improve coverage across currently recognized PCV4 lineages and reduce the risk of mutation‐associated detection failure as the virus continues to evolve.

In this study, we developed a *Rep*‐targeted SYBR Green I real‐time qPCR assay to screen clinical porcine samples collected across China, characterizing the temporal, geographic, and production‐stage patterns of PCV4 infection. To elucidate the evolutionary dynamics of PCV4, we constructed a *Cap* gene phylogeny using newly sequenced and publicly available isolates. Bayesian phylodynamic and phylogeographic analyses were employed to estimate the evolutionary timescales and reconstruct spatial dispersal patterns among sampled locations. By integrating selection pressure analysis, epitope prediction, and structural mapping, we prioritized candidate sites of adaptive variation for future functional validation.

## 2. Materials and Methods

### 2.1. Sample Collection

Between January 2022 and November 2025, a total of 1920 tissue samples covering 29 provinces in China were obtained from the Animal Disease Diagnosis Center, Huazhong Agricultural University. The samples were derived from aborted fetuses, piglets, nursery pigs, fattening pigs, and sows and included heart, liver, spleen, lung, kidney, intestine, brain, lymph node, and placental tissues from 1920 pigs. Pooled tissue homogenates were prepared for nucleic acid extraction. Samples from symptomatic and asymptomatic pigs within affected commercial herds were included for descriptive comparison.

### 2.2. Sample Treatment and Nucleic Acid Extraction

Tissue samples were dissected into small pieces and transferred into sterile 2 mL centrifuge tubes containing 1 mL of phosphate‐buffered saline (PBS). Homogenization was performed at 60 Hz for 6 min using a tissue grinder. To facilitate viral release, the homogenates were subjected to three freeze‐thaw cycles at −80°C. Subsequently, the samples were centrifuged at 12,000 × *g* for 10 min, and the supernatants were collected and stored at −80°C. Viral nucleic acids were extracted using a Vazyme FastPure Viral DNA/RNA Mini Kit (Vazyme Biotech Co., Ltd., Nanjing, China, Cat. No. RM201‐02) following the manufacturer’s protocol. The nucleic acids were eluted in 50 μL of nuclease‐free water and stored at −80°C until use.

### 2.3. Primer Design

A total of 85 PCV4 sequences were retrieved from GenBank and aligned to identify conserved regions within the *Rep* gene. Multiple sequence alignment were performed using the MUSCLE algorithm implemented in MEGA 12 [15]. Based on the alignment, primers targeting a highly conserved region of the *Rep* gene were designed using Primer Premier 5.0. Candidate primers were evaluated for GC content, melting temperature, and the potential formation of hairpins, self‐dimers, and heterodimers. Predefined exclusion criteria were applied to reduce nonspecific amplification and primer‐dimer fluorescence: candidate primers were excluded if the predicted Gibbs free energy (Δ*G*) was ≤−5.0 kcal/mol for self‐dimers or heterodimers or ≤−3.0 kcal/mol for hairpins or if more than three consecutive complementary nucleotides were present at the 3′ termini. The selected forward and reverse primers were each 20 nucleotides long, with a GC content of 55.0% and a predicted melting temperature of 59.0°C. The primer pair amplified a 129‐bp fragment corresponding to nucleotide positions 181–309 of PCV4 strain HNU‐AHG1‐2019 (GenBank Accession No. MK986820.1).

Primer specificity was assessed in silico using the NCBI Primer‐BLAST tool against the comprehensive nucleotide collection (nr/nt) database. To ensure host background specificity, the search was explicitly restricted to the *Sus scrofa* taxonomy (taxid:9823) to exclude potential amplification of host genomic sequences. Additionally, candidate sequences were cross‐referenced against the complete genomes of common porcine viral pathogens in the GenBank database. No relevant nontarget amplification was predicted. The conservation of the two primer‐binding sites was further examined across the alignment of 85 PCV4 sequences, demonstrating a high degree of conservation at both binding regions. The complete 20‐nucleotide binding regions of both PCV4‐*Rep*‐F and PCV4‐*Rep*‐R were conserved in all 85 sequences. Primers were synthesized by Beijing Tsingke Biotech Co., Ltd. (Beijing, China), and their detailed characteristics are presented in Table 1.

**Table 1:** Primers used for PCV4 detection.

Name	Sequence 5′–3′	Primer length (bp)	Position	Tm (°C)	GC content (%)	Amplification size (bp)
PCV4‐*Rep*‐F	AGTACGCTGTTGTCGGGAAG	20	181–200	59.0	55.0	129
PCV4‐*Rep*‐R	GCTCAATATGGCTCCGCTCT	20	290–309	59.0	55.0	

### 2.4. Preparation of Standard Plasmid Controls

To construct the standard plasmid, a 129 bp PCV4 *Rep* target fragment corresponding to nucleotide positions 181–309 of the reference sequence MK986820.1 was amplified from a laboratory‐maintained plasmid containing the corresponding PCV4 sequence. The cloning primers are listed in Supporting Information 1. In parallel, the pET28a vector backbone was amplified using the corresponding vector‐specific primers. The amplified pET28a vector backbone and *Rep* fragment were digested with SacI and SalI and subsequently ligated using T4 DNA ligase (Thermo Fisher Scientific; Cat. No. EL0011). The ligation mixture was transformed into chemically competent *Escherichia coli* DH5α cells (TOLOBIO, Beijing, China; Cat. No. CC96102‐02). Colonies identified as positive were cultured at 37°C with shaking at 220 rpm for 12–16 h, followed by extraction of the recombinant plasmid DNA using a HiSpeed Plasmid Mini Kit (QIAGEN, Hilden, Germany) and subjected to Sanger sequencing by Beijing Tsingke Biotech Co. (Beijing, China) to verify the identity, integrity, and orientation of the *Rep* insert. The verified recombinant plasmid was designated pET28a‐PCV4‐*Rep*. Additional details of plasmid construction, including the cloning strategy, primer sequences, and sequencing chromatogram, are provided in Supporting Information 1. The concentration of pET28a‐PCV4‐*Rep* was determined using a NanoDrop 2000 spectrophotometer (Thermo Fisher Scientific, Waltham, MA, USA), and the plasmid copy number was calculated using the following equation: copy number (copies/µL) = [6.02 × 10^23^ × plasmid concentration (ng/µL) × 10^−9^]/[plasmid length (bp) × 660]. Finally, 10‐fold serial dilutions of the recombinant plasmid were prepared using nuclease‐free water to generate the standards for subsequent qPCR analysis.

### 2.5. Optimization of Reaction Conditions

Primer concentrations of 0.1, 0.2, 0.4, 0.6, 0.8, 1.0, and 1.2 µM and annealing temperatures of 55, 57, 58, 59, 61, 63, 64, and 65°C were evaluated using a two‐factor matrix design. Each temperature–concentration combination was tested in three technical replicates using a fixed concentration of the standard plasmid (1.17 × 10^6^ copies/µL), together with a corresponding no‐template control (NTC). Mean Ct values, standard deviations, melt‐peak temperatures, NTC outcomes, and the presence or absence of nonspecific amplification or primer‐dimer formation were recorded for each condition. The final condition was selected as the lowest primer concentration that produced a consistently low Ct value, low replicate variability, a single PCV4‐specific melt peak, and no detectable amplification in the corresponding NTC.

### 2.6. Establishment of Standard Curves

To establish the standard curve, the recombinant plasmid pET28a‐PCV4‐*Rep* was serially diluted 10‐fold to obtain eight concentrations ranging from 1.17 × 10^9^ to 1.17 × 10^2^ copies/µL. Each concentration was tested in three technical replicates, with nuclease‐free water included as the NTC. The standard curve was generated by plotting the Ct values against the log_10_‐transformed initial template copy numbers.

### 2.7. Specificity, Sensitivity, and Repeatability

To evaluate the analytical specificity of the developed qPCR assay, cross‐reactivity was assessed using a panel of common porcine viral pathogens. The PCV4‐positive control was a sequence‐verified pET28a‐PCV4‐*Rep* standard plasmid containing the 129‐bp *Rep* target fragment, corresponding to nucleotide positions 181–309 of the PCV4 reference sequence (GenBank: MK986820.1). Positive control materials for PCV2, (FJ598044.1), PCV3 (MG546667.1), porcine reproductive and respiratory syndrome virus (PRRSV, EU860248.1), porcine epidemic diarrhea virus (PEDV, KT021232.1), porcine parvovirus (PPV, AY789532.1), and pseudorabies virus (PRV, AF306511.1) were sourced from archived pathogen‐positive samples in our laboratory. The classical swine fever virus (CSFV, HM175885.1) positive control was prepared from a commercial live‐attenuated vaccine. The identities of all non‐PCV4 control materials were confirmed via whole‐genome sequencing and sequence alignment; the accession numbers in parentheses indicate the corresponding GenBank sequences identified through this comparison. For testing, DNA extracted from the PCV2, PCV3, PPV, and PRV samples was used directly, whereas RNA extracted from the PRRSV, PEDV, and CSFV samples was reverse‐transcribed into cDNA. Each control template was tested in triplicate, with nuclease‐free water serving as the NTC.

To estimate the LOD95% independently of the standard curve experiment, the pET28a‐PCV4‐*Rep* standard plasmid was diluted to 10, 8, 6, and 4 copies/µL. At each concentration, 20 technical replicate reactions were tested. Each replicate was classified as detected or not detected according to the predefined amplification and melt curve criteria. A reaction was classified as detected when the Ct value ≤40 and a single melting peak within 85.5 ± 0.5°C were observed. Reactions that did not meet both criteria were classified as not detected. Nondetected reactions were retained as binary nondetection outcomes and were not assigned an artificial Ct value. The replicate‐level binary detection outcomes across the concentration gradient were analyzed using probit regression in SPSS software (SPSS Inc., Chicago, IL, USA). The LOD95% was defined as the concentration predicted to yield a 95% probability of detection, and the corresponding 95% confidence interval (CI) was estimated from the fitted model.

To assess assay precision, intra‐assay repeatability and inter‐assay precision were initially evaluated at three standard plasmid concentrations (1.17 × 10^7^, 1.17 × 10^6^, and 1.17 × 10^5^ copies/µL). At each concentration, intra‐assay repeatability was assessed using three technical replicates within a single run, whereas inter‐assay precision was assessed across three independent runs performed on different days. The assay performance near the detection limit was further evaluated at 4.11, 8.22, 16.44, and 24.66 copies/µL, corresponding to ~0.5×, 1×, 2×, and 3× the estimated LOD95%, respectively. For intra‐assay repeatability, 10 technical replicate reactions per concentration were tested within a single run. For inter‐assay precision, a total of 10 reactions per concentration were distributed across three independent runs.

### 2.8. Agreement Between the SYBR Green I and TaqMan qPCR Assays in Clinical Specimens

To evaluate agreement between the developed SYBR Green I qPCR assay and the reference TaqMan qPCR assay [16] in clinical samples, 40 samples were selected from the tested samples described in Section 2.1. Each specimen originated from a different pig and a different farm. Thirty PCV4‐positive samples were purposively selected according to their reference TaqMan qPCR Ct values to represent three predefined viral load groups: high viral load (Ct ≤ 20, *n* = 10), intermediate viral load (20 < Ct ≤ 25, *n* = 10), and lower viral load (25 < Ct ≤ 30, *n* = 10). An additional 10 samples previously classified as PCV4‐negative by the reference TaqMan qPCR assay were included as negative samples. Nucleic acids were independently extracted from each specimen using the extraction procedure described in Section 2.2 and subsequently tested in parallel using the developed SYBR Green I qPCR assay. For the SYBR Green I assay, samples yielding amplification within the predefined Ct cutoff together with the characteristic PCV4‐specific melting peak were considered positive.

Qualitative agreement between the two assays was evaluated using overall percent agreement and Cohen’s kappa coefficient. Positive agreement was additionally examined within the high, intermediate, and lower viral load groups. For quantitative comparison, the association between Ct values was assessed using Spearman’s correlation analysis and linear regression, with the TaqMan qPCR Ct value as the independent variable and the SYBR Green I qPCR Ct value as the dependent variable. The agreement between paired Ct values was further evaluated using Bland–Altman analysis. The Ct difference was calculated as the SYBR Green I qPCR Ct minus the TaqMan qPCR Ct, and the 95% limits of agreement were calculated as the mean difference ±1.96 standard deviations. Potential proportional bias was assessed by linear regression of the paired differences against the corresponding mean Ct values. A two‐sided *p* value <0.05 was considered statistically significant.

### 2.9. Clinical Sample Detection

All submitted samples were tested using the optimized assay to determine PCV4 positivity and describe its spatial and temporal distribution in China from 2022 to 2025.

### 2.10. PCV4 *Cap* Gene Phylogenetic Reconstruction

To evaluate the evolutionary dynamics of PCV4, a *Cap* gene dataset was assembled [17], comprising 82 global reference sequences from GenBank and 15 clinical sequences generated in this study Supporting Information 2 Table S1. The *Cap* gene was selected because substantially more geographically and temporally annotated *Cap* sequences than complete genomes were available in public databases, permitting broader comparative coverage. Multiple sequence alignment were performed using MAFFT v7.5 [18]. Maximum likelihood (ML) phylogenetic reconstruction was conducted in IQ‐TREE 2.0 [19]. The optimal nucleotide substitution model, K3Pu + G4, was selected using ModelFinder [20]. Branch support was assessed with 1000 ultrafast bootstrap replicates [21]. The resulting ML trees were visualized and annotated using the Chiplot platform [22].

### 2.11. Phylodynamic Analysis of the PCV4 *Cap* Gene

An initial global *Cap* gene dataset comprising 92 sequences was assembled for the Bayesian phylodynamic analysis. Sequences were annotated with sampling dates and locations, aligned, and automatically trimmed using Gblocks [23]. Before molecular clock analysis, the alignment was screened for potential recombination using RDP5 and GARD. Although RDP5 returned two tentative algorithm‐specific signals, these were flagged as potentially attributable to nonrecombinant evolutionary processes and were not independently supported by GARD. Therefore, no convincing evidence of recombination was considered to be present in the alignment. Accordingly, no sequences were excluded on the basis of the recombination screening. Independently of the recombination analysis, three sequences generated in this study were excluded from the Bayesian dataset because of insufficient sequence length/alignment coverage or high sequence redundancy with other study‐derived sequences. The final global dataset used for BEAST analysis therefore comprised 89 *Cap* gene sequences Supporting Information 3 Table S2.

Time‐calibrated phylogenies were inferred from the final 89‐sequence dataset using the GTR + Γ substitution model and a strict molecular clock. A Bayesian Skyline coalescent prior was used to reconstruct changes in relative effective population size through time without imposing a predefined parametric demographic growth model. Two independent MCMC runs were performed using the same 89‐sequence dataset and identical model settings but different random‐number seeds. Each chain was run for 200 million generations, with parameters and trees being sampled every 20,000 generations. The two runs were initially assessed separately in Tracer v1.7. Inspection of the trace plots showed that the posterior, likelihood, and major model parameters had reached stable stationary distributions before the 10% cutoff; therefore, the initial 10% of each chain was discarded as a conservative and consistently applied burn‐in. The two runs yielded comparable posterior estimates with overlapping 95% HPD intervals, and the ESS values of all key parameters exceeded 200 in each independent run. After these convergence criteria were satisfied, the post‐burn‐in log and tree files were combined using LogCombiner for the final posterior estimation. Run‐specific posterior estimates and convergence diagnostics are reported in Supporting Information 4 Table S3A, and the corresponding estimates from the combined post‐burn‐in posterior distribution are reported in Supporting Information 4 Table S3B.

Maximum clade credibility (MCC) trees were generated from the combined posterior tree distribution using TreeAnnotator and visualized in FigTree. SpreaD3 was used to calculate Bayes factor (BF) values and posterior probabilities (PPs) for discrete geographic transitions. Geographic links with BF > 3 and PP > 0.3 were retained as statistically supported, model‐inferred associations. These inferred geographic links were subsequently mapped using ArcGIS Pro.

### 2.12. Selection Pressure and Protein Structure Prediction

Selection pressure on the PCV4 Cap protein coding sequence was explored using Datamonkey [24] with SLAC, FEL, MEME, and FUBAR [25]. Sites meeting*p* < 0.1 in SLAC, FEL, or MEME, or PP >0.9 in FUBAR, were considered candidate positively selected sites. Results were interpreted cautiously because these thresholds are permissive, and SLAC detected no candidate positively selected sites. The three‐dimensional structure of the PCV4 Cap protein (reference sequence MK986820) was predicted using AlphaFold3. Modeling was focused on residues 24–228 because of the predicted disorder at the N‐terminus. Confidence was evaluated using pTM, pLDDT, and the PAE matrix. SWISS‐MODEL was used to identify a structural homolog for a 60‐mer virus‐like particle, structural alignments were performed in PyMOL v2.6, and candidate sites were mapped onto the predicted monomer.

### 2.13. Prediction of Linear B‐Cell Epitopes and T‐Cell Epitopes on the Cap Protein and Structural Mapping

In this study, B‐cell and T‐cell epitopes of the Cap protein were predicted using the Immune Epitope Database (IEDB) server based on its amino acid sequence. MHC class I‐binding peptides (SLA‐1 04:01, SLA‐1 07:01, SLA‐2 04:01, and SLA‐3 04:01) were predicted for selected swine leukocyte antigen alleles to identify candidate T‐cell epitopes. Linear B‐cell epitopes were predicted to identify candidate antibody‐recognition regions. Structural mapping was performed using PyMOL (v2.6).

### 2.14. Statistical Analysis

Statistical analyses were performed using GraphPad Prism 9.5 (GraphPad Software, USA). Positivity rates and their 95% CIs were calculated using the Wilson method. Differences in positivity rates among years and production stages were assessed using Pearson’s chi‐square test. Temporal trends in positivity rates were evaluated using the Cochran–Armitage trend test. *p* < 0.05 was considered statistically significant. Spatial distribution and temporal patterns of infection were visualized using ArcGIS Pro (Esri, USA). Viral loads were log_10_‐transformed before statistical analysis. Differences in viral loads between symptomatic and asymptomatic pigs across production stages were analyzed using two‐way ANOVA, followed by Šídák’s multiple comparisons test. Adjusted *p* values <0.05 were considered statistically significant.

## 3. Results

### 3.1. Analytical Performance of the PCV4 qPCR Assay

Sanger sequencing completely covered the 129 bp PCV4 *Rep* fragment. The mean Phred quality score was 47.1. All 129 bases had quality scores ≥Q20, and 127/129 bases (98.45%) had quality scores ≥Q30. Alignment with the intended PCV4 *Rep* target confirmed 100% nucleotide identity across the complete insert, with no substitutions, insertions, or deletions. The insert orientation was also consistent with the expected construct (Supporting Information 1). These results confirmed the successful construction of the recombinant standard plasmid, designated pET28a‐PCV4‐*Rep*.

Based on the orthogonal optimization results, the optimal annealing temperature was established at 63°C, with a final primer concentration of 400 nM. The final 20‐µL amplification reaction consisted of 10 µL of qPCR SYBR Green Master Mix (Yeasen, 10167ES50), 400 nM of each primer, 2 µL of the DNA template, and nuclease‐free water to bring the volume to 20 µL (Figure 1A). All NTCs yielded no amplification, confirming the specificity of the assay. The thermocycling conditions were programed as follows: an initial predenaturation step at 95°C for 10 min, followed by 40 cycles of denaturation at 95°C for 10 s, and annealing/extension at 63°C for 1 min.

**Figure 1:**
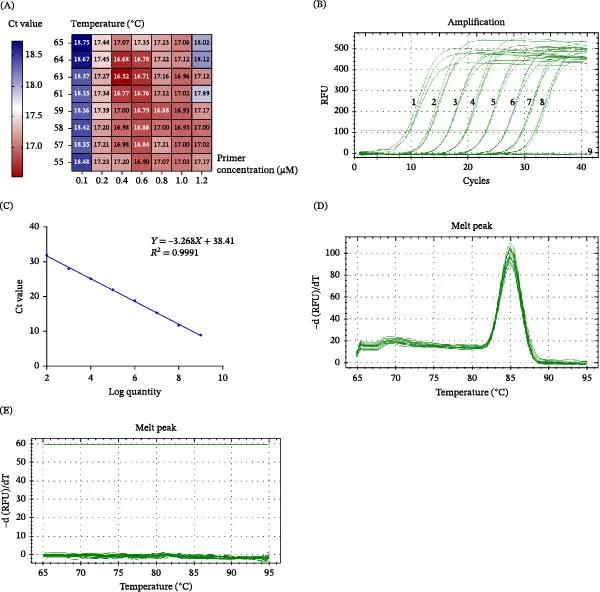
Optimization and analytical performance of the PCV4 SYBR Green I qPCR assay. (A) Mean Ct‐value matrix obtained during optimization of annealing temperature and primer concentration. Each value represents the mean of three technical replicates. (B) The eight 10‐fold serial dilutions of the standard plasmid, ranging from 1.17 × 10^9^ to 1.17 × 10^2^ copies/µL, generated clearly separated amplification curves, with close clustering among the three technical replicates at each concentration. In descending concentration order, the corresponding mean Ct values were 8.93, 11.71, 15.33, 18.85, 21.98, 25.07, 28.02, and 31.87, respectively. No detectable amplification was observed in the NTC. (C) Standard curve generated from serial plasmid dilutions. (D) Melting‐curve profiles of PCV4‐positive reactions across the plasmid dilution series. (E) Representative melting‐curve profiles of the non‐PCV4 pathogen controls, the NTC, and representative PCV4‐negative clinical specimens.

The optimization matrix showed comparable performance across several adjacent temperature–concentration combinations, with a single specific melt peak and no detectable NTC amplification under each of these conditions. Among them, 63°C with 400 nM of each primer yielded the numerically lowest mean Ct value (16.52 ± 0.22; *n* = 3), corresponding to the earliest threshold crossing while maintaining a single melt peak at ~85.5°C. Increasing the primer concentration above 400 nM provided no further improvement in Ct values. Therefore, 63°C and 400 nM of each primer were selected as the final conditions.

The recombinant plasmid pET28a‐PCV4‐*Rep* was serially diluted 10‐fold from 1.17 × 10^9^ to 1.17 × 10^2^ copies/µL. Each concentration was tested in triplicate (Figure 1B). The standard curve was generated by plotting the Ct values against the log_10_‐transformed initial template copy numbers. Linear regression analysis of the standard curve yielded the equation *Y* = −3.268*X* + 38.41 (*R* ^2^ = 0.9991), corresponding to an amplification efficiency of 102.3% (Figure 1C). Product specificity was further supported by melt curve analysis. All PCV4‐positive replicates generated a single distinct melting peak, with a mean melting temperature of 85.5 ± 0.5°C. No additional peaks indicative of nonspecific amplification or primer‐dimer formation were observed in any positive replicate (Figure 1D). Representative melting curves for the non‐PCV4 pathogen panel, including PCV2, PCV3, PRRSV, PEDV, CSFV, PPV, and PRV, together with the NTC and representative PCV4‐negative clinical specimens, are shown in Figure 1E. None of these negative controls produced a PCV4‐specific melting peak.

### 3.2. Specificity, Sensitivity, and Reproducibility

The developed qPCR assay exhibited high analytical specificity for PCV4, with no cross‐reactivity observed against other common porcine pathogens, including PCV2, PCV3, PRRSV, PEDV, CSFV, PPV, and PRV (Figure 2A). Among the 20 technical replicate reactions tested at each concentration, PCV4 was detected in 20/20 reactions at 10 copies/µL, 19/20 at 8 copies/µL, 14/20 at 6 copies/µL, and 10/20 at 4 copies/µL. Probit regression of these replicate‐level binary detection outcomes estimated an LOD95% of 8.22 copies/µL (95% CI: 7.08–11.22 copies/µL; Figure 2B).

**Figure 2:**
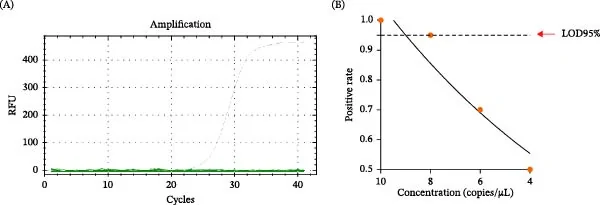
(A) Specificity of the developed SYBR Green I qPCR assay for PCV4. (B) LOD95% of the developed SYBR Green I qPCR assay for PCV4.

For reproducibility, intra‐assay and inter‐assay variability were assessed using three standard plasmid concentrations (1.17 × 10^7^, 1.17 × 10^6^, and 1.17 × 10^5^ copies/µL). The coefficients of variation (CVs) for the Ct values ranged from 0.46% to 1.26% for intra‐assay replicates and from 0.93% to 0.97% for inter‐assay replicates (Table 2). All CV values were below 1.5%, indicating good repeatability and reproducibility.

**Table 2:** Repeatability and reproducibility evaluations of the PCV4 SYBR Green I real‐time PCR assay.

Plasmid concentration (copies/µL)	Intra‐assay repeatability		Inter‐assay reproducibility	
	Mean ± SD	CV (%)	Mean ± SD	CV (%)
1.17 × 10^7^	18.32 ± 0.23	1.26	18.34 ± 0.17	0.93
1.17 × 10^6^	23.61 ± 0.11	0.46	23.92 ± 0.23	0.96
1.17 × 10^5^	28.76 ± 0.36	1.26	28.89 ± 0.28	0.97

Additional repeatability and reproducibility testing was performed at concentrations near the LOD95%, including ~0.5×, 1×, 2×, and 3× the estimated LOD95%. At concentrations of 1 × LOD95% and above, all intra‐ and inter‐assay replicates yielded detectable amplification, with Ct CVs ranging from 0.56% to 1.25%. In contrast, detection became less consistent at 0.5 × LOD95%, with 4/10 intra‐assay replicates and 5/10 inter‐assay replicates yielding detectable amplification. Among the positive reactions at 0.5 × LOD95%, the Ct CVs were 1.76% and 1.50% for the intra‐ and inter‐assay evaluations, respectively. These results indicate increased stochastic nondetection below the estimated LOD95%, while reproducibility remained stable at concentrations at or above the LOD95% (Table 3).

**Table 3:** Repeatability and inter‐assay precision of the PCV4 SYBR Green I qPCR assay near the LOD95%.

Plasmid concentration (copies/µL)	Intra‐assay repeatability			Inter‐assay reproducibility		
	Positive /total	Mean ± SD	CV (%)	Positive /total	Mean ± SD	CV (%)
4.11 (0.5 × LOD95%)	4/10	39.03 ± 0.69	1.76	5/10	39.34 ± 0.59	1.50
8.22 (1 × LOD95%)	10/10	35.41 ± 0.22	0.61	10/10	35.47 ± 0.20	0.57
16.44 (2 × LOD95%)	10/10	34.68 ± 0.24	0.69	10/10	34.58 ± 0.43	1.25
24.66 (3 × LOD95%)	10/10	33.38 ± 0.19	0.56	10/10	33.62 ± 0.25	0.76

### 3.3. Agreement Between the Different Assays in Clinical Samples

As shown in Table 4, the developed SYBR Green I qPCR assay showed complete qualitative agreement with the reference TaqMan qPCR assay [16] across the evaluated sample panel. All 10 specimens in each of the high‐ (Ct ≤ 20), intermediate‐ (20 < Ct ≤ 25), and lower viral load (25 < Ct ≤ 30) groups were consistently detected as positive by both assays, whereas all 10 negative clinical specimens tested negative by both assays. No discordant results were observed, resulting in an overall agreement of 100% (40/40) and a Cohen’s kappa coefficient of 1.00 (*p* < 0.001).

**Table 4:** Qualitative agreement between the developed SYBR Green I qPCR assay and the reference TaqMan qPCR assay in 30 naturally PCV4‐positive and 10 PCV4‐negative clinical specimens.

SYBR Green qPCR	TaqMan qPCR		Total
	+	−	
Ct ≤ 20	10	0	10
20 < Ct ≤ 25	10	0	10
25 < Ct ≤ 30	10	0	10
—	0	10	10
Total	30	10	40

*Note:* Viral‐load groups were defined according to Ct values obtained using the reference TaqMan qPCR assay. “+” and “−” indicate positive and negative detection results, respectively. The evaluated panel consisted of 30 naturally PCV4‐positive clinical specimens and 10 PCV4‐negative clinical specimens. No standard plasmid was added to the specimens used in this agreement analysis.

Quantitative comparison of paired Ct values from the 30 positive specimens showed a very strong correlation between the two assays (Spearman’s *ρ* = 0.9978, *p* < 0.001). Linear regression yielded the equation *Y* = 1.0021*X* − 0.0571 (*R* ^2^ = 0.9995), where *X* represents the TaqMan qPCR Ct value and *Y* represents the SYBR Green I qPCR Ct value (Figure 3). Because correlation measures association rather than agreement, agreement between the paired Ct measurements was further evaluated using Bland–Altman analysis. The mean Ct difference, calculated as the SYBR Green I qPCR Ct minus the TaqMan qPCR Ct, was −0.011 cycles, with estimated 95% limits of agreement ranging from −0.197 to 0.174 cycles. All 30 paired measurements fell within these limits. Regression of the paired differences against the corresponding mean Ct values showed no statistically significant evidence of proportional bias (slope = 0.0023, *p* = 0.575).

**Figure 3:**
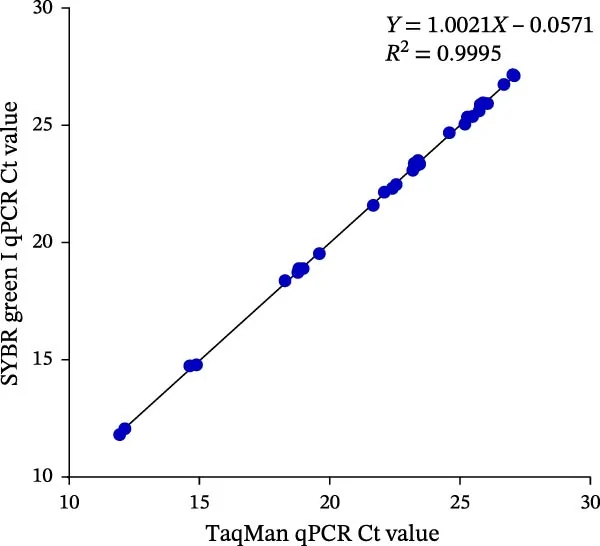
Correlation of Ct values obtained using the SYBR Green I and TaqMan qPCR assays.

### 3.4. Clinical Positivity and Spatiotemporal Distribution of PCV4

Screening of 1920 diagnostic samples collected between 2022 and 2025 revealed an overall PCV4 positivity rate of 5.00% (96/1920; 95% CI: 4.11%–6.07%). Over the 2022–2025 surveillance period, the annual PCV4 positivity rates were 3.07% in 2022, 4.70% in 2023, 5.09% in 2024, and 8.56% in 2025 (Figure 4). Within this diagnostic submission dataset, a statistically significant increase in PCV4 positivity was observed from 2022 to 2025 (*χ* ^2^trend = 11.60, *p* = 0.00066).

**Figure 4:**
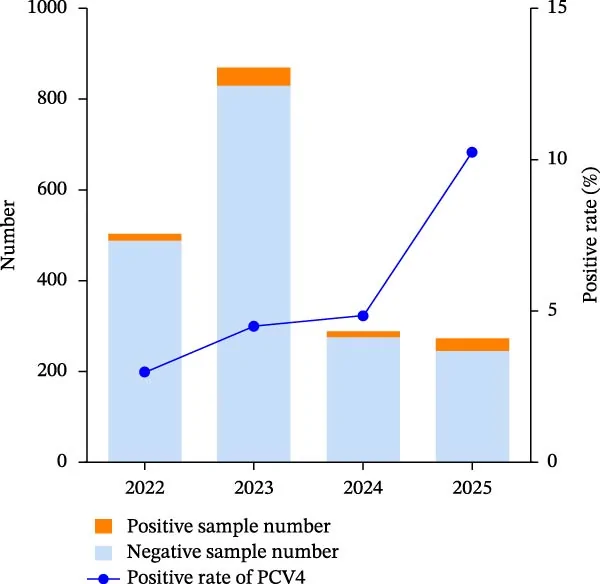
Annual positivity of PCV4 from the year of 2022 to 2025.

Although an increasing annual PCV4 positivity trend was observed among the tested samples, unequal sampling intensity across regions limits direct comparison of the regional positivity proportions and warrants cautious interpretation of the spatial patterns. Provinces were grouped into seven geographical regions according to standard administrative divisions (Table 5). The highest positivity rate was observed in Northeast China (7.76%, 9/116; 95% CI: 4.07%–14.13%), closely followed by South China (7.60%, 19/250; 95% CI: 4.90%–11.62%) and Eastern China (5.49%, 18/328; 95% CI: 3.48%–8.53%), while the lowest positivity rate was observed in North China (2.49%, 5/201; 95% CI: 1.07%–5.69%), followed by Central China (3.85%, 22/572; 95% CI: 2.55%–5.76%). The province‐level distribution of PCV4‐positive samples is shown in Figure 5. Because regional sample sizes were uneven, these differences describe the submitted sample set and cannot establish regional population prevalence or localized clustering.

**Figure 5:**
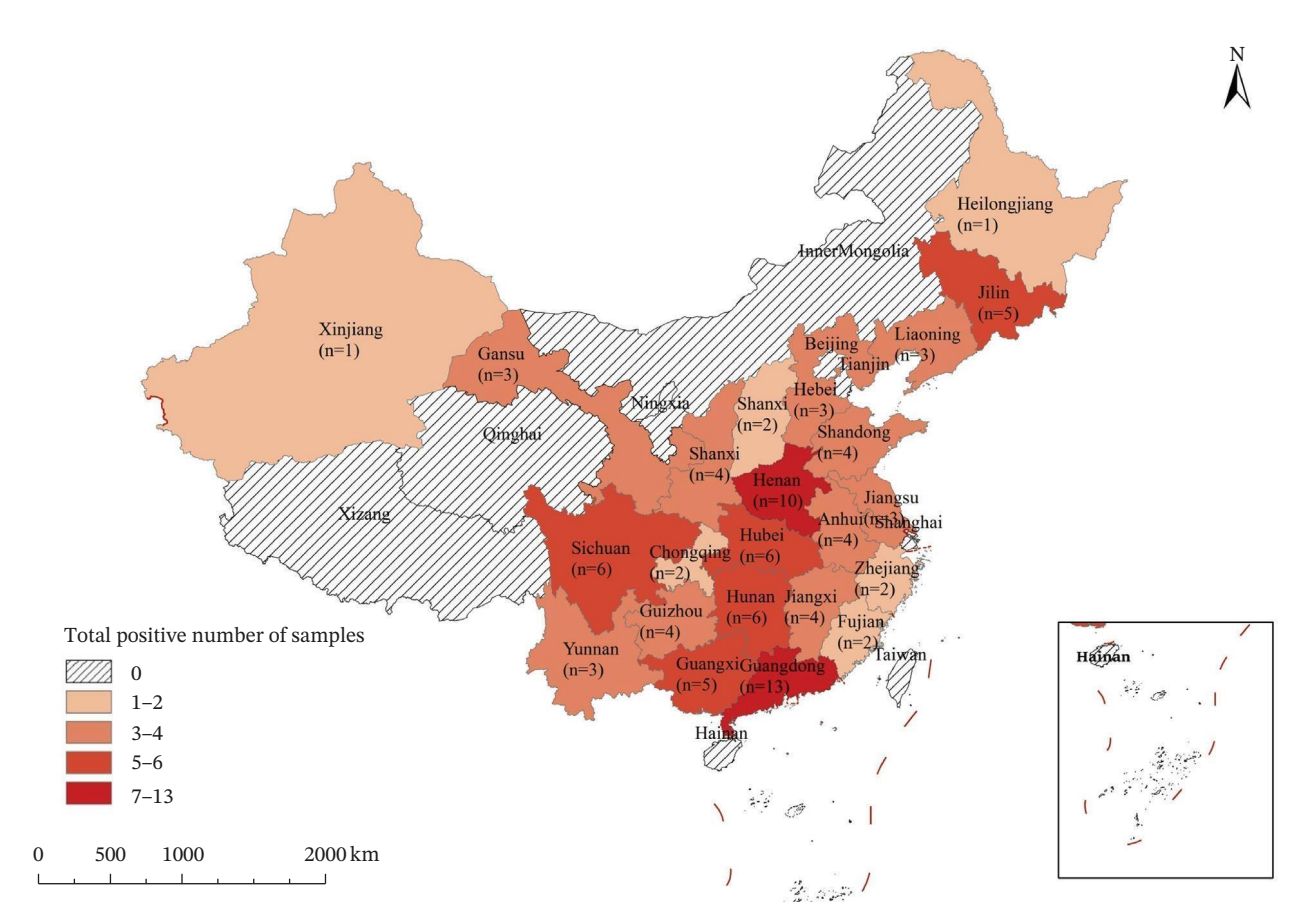
Province‐level regional distribution of PCV4‐positive samples in China.

**Table 5:** Regional distribution of PCV4‐positive samples in China.

Region	Number of PCV4 positive samples	Number of samples	Positivity rate
North China	5	201	2.49% (5/201)
Northeast China	9	116	7.76% (9/116)
Eastern China	18	328	5.49% (18/328)
Central China	22	572	3.85% (22/572)
South China	19	250	7.60% (19/250)
Southwest China	15	279	5.38% (15/279)
Northwest China	8	174	4.60% (8/174)
Total	96	1920	5.00% (96/1920)

### 3.5. PCV4 Viral Loads and Positivity Across Production Stages

The quantitative analysis showed a highly skewed distribution of viral loads, ranging from 3.39 × 10^2^ to 1.73 × 10^8^ copies/µL (mean: 4.64 × 10^6^ copies/µL). Among the positive cases, 12.5% (12/96, 95% CI: 7.26%–20.71%) had high viral loads exceeding 1.0 × 10^5^ copies/µL.

To compare PCV4 positivity across production stages, 1920 clinical samples were categorized into five groups: piglets (≤21 days), nursery pigs (22–70 days), fattening pigs (>70 days), sows, and aborted fetuses. The positivity rates across production stages are summarized in Table 6. Sows exhibited the highest detection rate at 7.89% (6/76, 95% CI: 3.65%–16.37%), followed by aborted fetuses at 6.27% (20/319, 95% CI: 4.09%–9.52%). Lower rates were observed in nursery pigs (4.98%, 14/281), piglets (4.68%, 40/854), and fattening pigs (4.10%, 16/390). Subsequent chi‐square analysis showed no statistically significant difference in PCV4 positivity across the five production stages (*p* > 0.05).

**Table 6:** Positivity of PCV4 in pigs across different production stages.

Production stages	Positive detections/total samples	Positive rate (%)
Piglets	40/854	4.68
Nursery pigs	14/281	4.98
Fattening pigs	16/390	4.10
Sows	6/76	7.89
Aborted fetuses	20/319	6.27
Total	96/1920	5.00

Within each production‐stage group, viral loads were further compared between symptomatic and asymptomatic pigs. Viral load distributions across production stages are shown in Figure 6A, and comparisons between symptomatic and asymptomatic pigs are shown in Figure 6B. Symptomatic piglets showed significantly higher viral loads than asymptomatic piglets (adjusted *p* = 0.0006), and symptomatic aborted fetuses also had significantly higher viral loads than asymptomatic aborted fetuses (adjusted *p* = 0.0044). No significant differences were observed in nursery pigs, fattening pigs, or sows.

**Figure 6:**
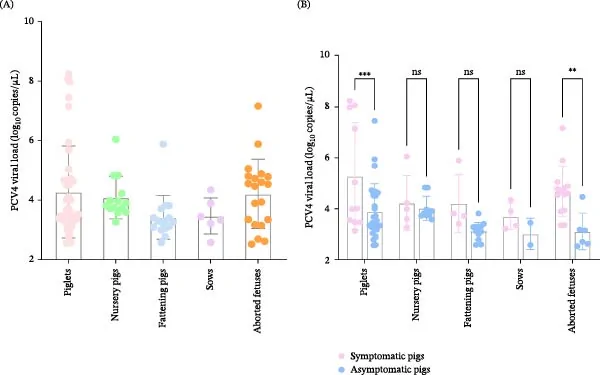
Analysis of PCV4 viral loads across different pig populations and clinical statuses. (A) Distribution of PCV4 viral loads in different pig populations. The scatter plot displays the detected levels of PCV4 viral DNA (log_10_ copies/µL) across five distinct sample categories: piglets (pink), fattening pigs (light blue), nursery pigs (green), aborted fetuses (purple), and sows (orange). (B) Comparison of PCV4 viral loads between symptomatic and asymptomatic pigs. Within the five aforementioned categories, this subfigure further compares the differences in PCV4 viral loads between pigs presenting with clinical symptoms (symptomatic pigs, pink circles) and those without (asymptomatic pigs, blue circles). ∗^∗^ *p* < 0.01,  ∗∗^∗^ *p* < 0.001; ns, not significant.

### 3.6. Phylogenetic Relationships and Host‐Associated Patterns

To examine the genetic relationships of contemporary PCV4 strains, we performed a phylogenetic analysis using the 15 *Cap* gene sequences obtained in this study and 82 reference strains. The resulting ML tree is shown in Figure 7.

**Figure 7:**
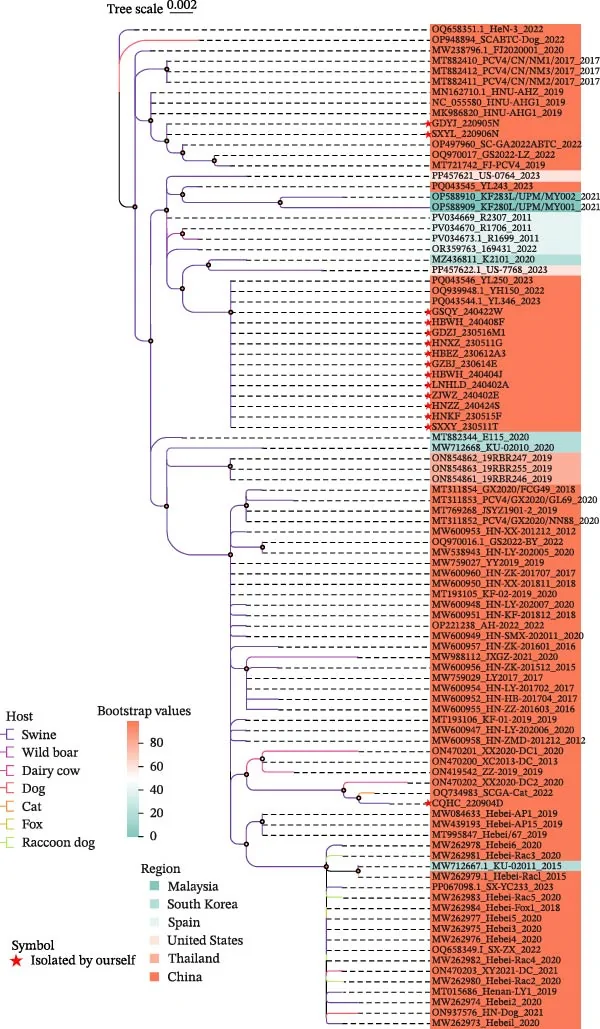
Maximum‐likelihood phylogenetic tree based on the PCV4 *Cap* gene.

The ML tree revealed that the circulating PCV4 strains clustered into previously described genotypes, with PCV4a identified as the predominant genotype in China. Phylogenetic analysis assigned the 15 novel PCV4 strains obtained in this study to two distinct clades. The vast majority (13 isolates) clustered within the predominant PCV4a branch, aligning closely with the bulk of domestic reference strains. The other two isolates, designated SXYL_220906N and GDYJ_220905N, were placed in the PCV4b branch.

Beyond genotypic classification, the phylogenetic tree suggested host‐associated mixing among available sequences. In terms of host diversity, the phylogenetic analysis incorporated not only sequences from domestic pigs but also isolates from various other mammals, including wild boars [26], dairy cows [17], dogs [27], foxes [28], cats [29], and raccoon dogs [28]. Notably, these sequences did not segregate into independent, host‐specific monophyletic clades. Instead, they were interspersed among porcine‐derived strains.

From a geographic perspective, international strains originating from Spain, the United States, South Korea, Malaysia, and Thailand were interspersed with Chinese sequences in the *Cap* gene tree. This topology suggests geographic and host‐associated mixing among the sampled sequences.

### 3.7. Recombination Screening

Recombination was screened by RDP5 and GARD. RDP5 identified two tentative recombination signals in the PCV4 *Cap* gene alignment. MaxChi, Chimaera, and 3Seq supported the first signal detected in ON470202 and a cluster of identical or near‐identical sequences, whereas only MaxChi supported the second signal detected in OP588909. However, both signals were identified by RDP5 as potentially attributable to evolutionary processes other than recombination. Meanwhile, GARD detected no statistically supported recombination breakpoint. Taken together, these findings were considered insufficient evidence for confirmed recombination. Therefore, the sequences associated with the two tentative RDP5 signals were retained in the final alignment used for the Bayesian analyses.

### 3.8. PCV4 *Cap* Gene‐Based Evolutionary Dynamics

The strict‐clock Bayesian analysis based on the final 89‐sequence *Cap* gene dataset estimated an evolutionary rate of 4.76 × 10^−4^ substitutions/site/year (95% HPD, 2.73 × 10^−4^ − 7.00 × 10^−4^) and a global tMRCA of ~1996.6 (95% HPD, 1980.5 − 2008.4). Two independent MCMC runs performed using identical model settings, but different random‐number seeds yielded comparable posterior distributions. After the removal of the initial 10% burn‐in from each run, the post‐burn‐in log and tree files were combined for final posterior estimation. All key parameters had ESS values >200 in each independent run and in the combined posterior distribution. Run‐specific posterior estimates and convergence diagnostics are presented in Supporting Information 4 Table S3A, and the corresponding combined estimates are presented in Supporting Information 4 Table S3B. The Bayesian Skyline reconstruction suggested a comparatively stable relative effective population size, followed by an apparent recent decline, with increased posterior uncertainty toward the most recent period (Figure 8).

**Figure 8:**
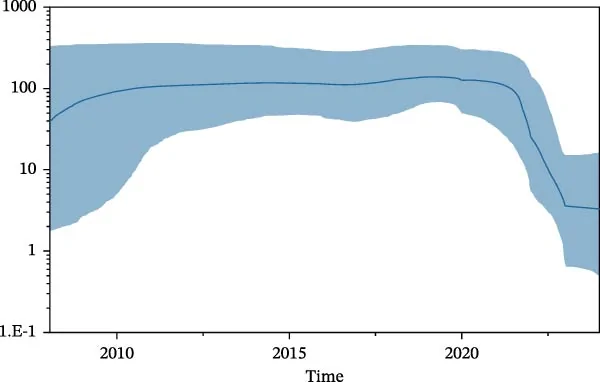
Bayesian Skyline reconstruction of the relative effective population size of PCV4 through time. The solid line represents the posterior median estimate, and the shaded area indicates the 95% highest posterior density interval.

### 3.9. Domestic Phylogeographic Analysis

To explore the geographic structure of available PCV4 *Cap* sequences within China, we performed a province‐level discrete phylogeographic reconstruction. The MCC tree showed geographically interspersed lineages (Figure 9A). BSSVS retained 13 model‐inferred geographic links under the selected BF and PP thresholds (Figure 9B). These links represent model‐based phylogenetic associations among sampled locations, not verified transmission routes. Their number and direction are sensitive to the small and uneven sequence counts per province.

**Figure 9:**
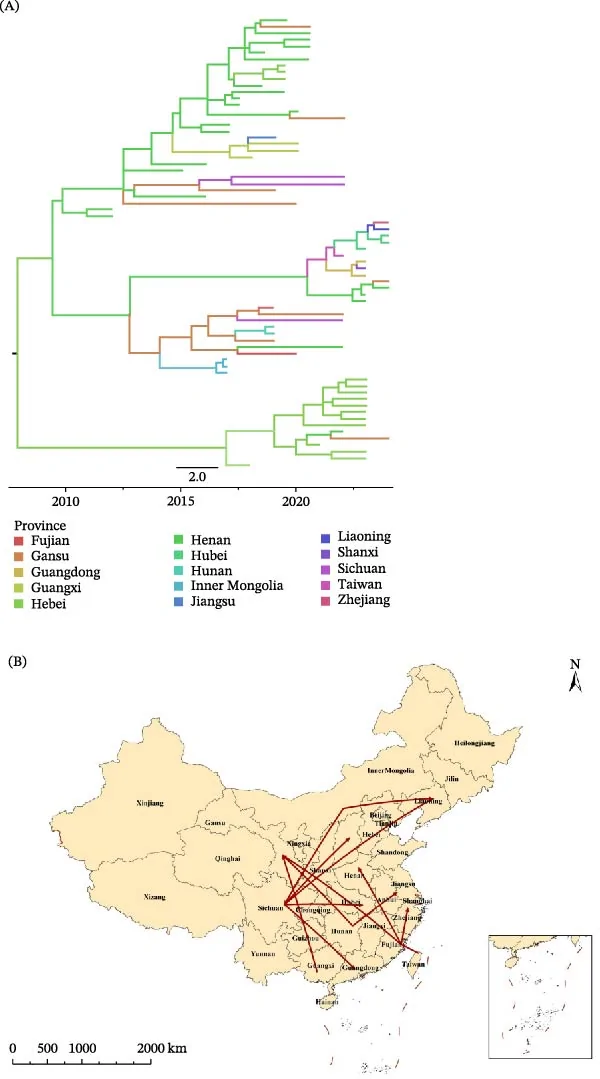
(A) Bayesian MCC tree for *Cap* gene of PCV4 in China. (B) Model‐inferred geographic links of PCV4 in China.

### 3.10. International Phylogeographic Analysis

In the global *Cap* gene dataset, Chinese sequences were numerically dominant, reflecting their much greater sampling density. Malaysian, Spanish, Thai, South Korean, and United States sequences occupied different phylogenetic positions (Figure 10), indicating genetic links among the sampled regions. Because of severe spatial sampling imbalance, these placements and any BSSVS‐derived model‐inferred geographic links must be interpreted cautiously and cannot identify a geographic origin, transmission direction, or primary dissemination hub.

**Figure 10:**
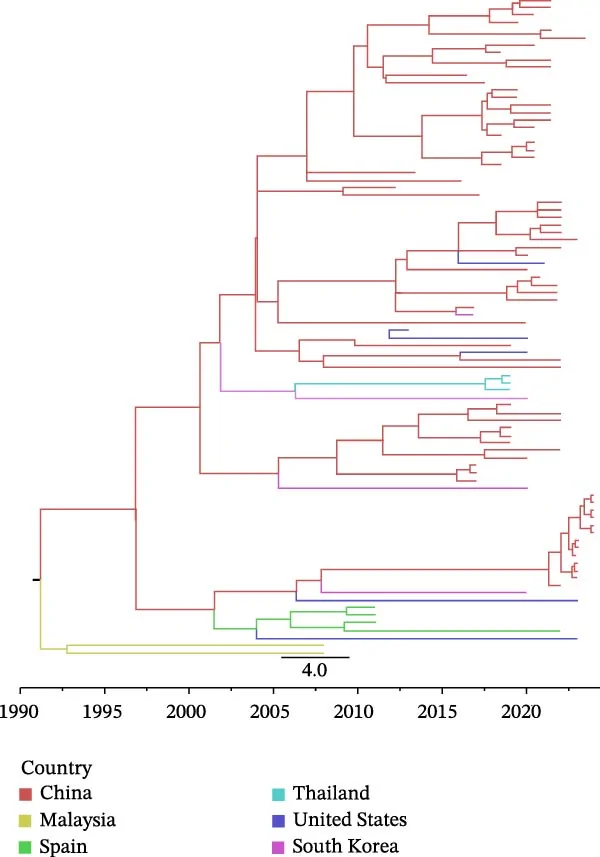
Bayesian MCC tree for *Cap* gene of PCV4 worldwide.

### 3.11. Selection Pressure and Protein Structure Mapping

Selection analyses indicated that most *Cap* codons were subject to purifying selection. Using the prespecified screening thresholds (MEME, FEL, and SLAC: *p* < 0.1; FUBAR: PP >0.9), MEME, FEL, and FUBAR identified three candidate positively selected sites (Ser27, Ser53, and Val98), whereas SLAC identified none (Table 7). These relatively permissive thresholds were used for exploratory candidate screening rather than the definitive identification of adaptive mutations. Accordingly, the three residues were interpreted as hypothesis‐generating candidates under positive selection rather than confirmed adaptive mutations. Their structural context was subsequently explored using an AlphaFold3‐predicted Cap model.

**Table 7:** Summary of candidate positively selected sites in the PCV4 *Cap* gene.

Gene	Method	Number of candidate positively selected sites	Candidate positively selected sites (codon position; *p*‐value or posterior probability)
PCV4 *Cap* gene	MEME	2	Ser53 (0.0688), Val98 (0.0953)
	FEL	1	Val98 (0.0919)
	FUBAR	1	Ser27 (0.9845)
	SLAC	0	—

*Note:* MEME/FEL/SLAC: *p* < 0.1; FUBAR: posterior probability >0.9.

The predicted *Cap* monomer had a pTM score of 0.87 (Figure 11A). The jelly roll core generally showed higher per‐residue confidence (pLDDT >70), whereas parts of the N‐terminal region had lower confidence (pLDDT <70), consistent with predicted local disorder. The three candidate sites were then positively selected and mapped onto a 60‐mer VLP model generated using the PCV2 *Cap* structure (PDB ID: 6OLA) as a template (Figure 11B). Structural proximity alone does not establish an effect on antigenicity or immune escape.

**Figure 11:**
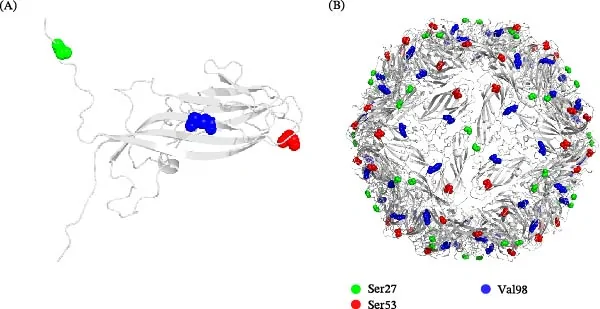
Structural modeling and spatial mapping of candidate positively selected sites on the PCV4 Cap protein. (A) PCV4 Cap monomer. The AlphaFold3‐predicted structure displays the characteristic viral jelly‐roll fold. Candidate positively selected sites (Ser27, Ser53, and Val98) are highlighted as colored spheres. (B) Assembled PCV4 capsid. Modeled using the PCV2 capsid (PDB ID: 6OLA) as a template, this 60‐mer icosahedral VLP illustrates the overall spatial distribution of the selected sites.

### 3.12. Epitope Prediction

Cross‐referencing the exploratory selection results with epitope predictions showed that the three candidate positively selected sites overlapped predicted epitope‐associated regions (Supporting Information 5 Table S4). Ser27 occurred within a predicted linear B‐cell epitope (residues 17–36), Ser53 within a predicted SLA‐I‐binding peptide (residues 51–60; rank <0.5%), and Val98 within an overlapping predicted B‐cell and SLA‐I‐binding region (residues 93–100; rank <0.5%). The structural mapping of predicted epitopes and candidate positively selected sites is shown in Figure 12.

**Figure 12:**
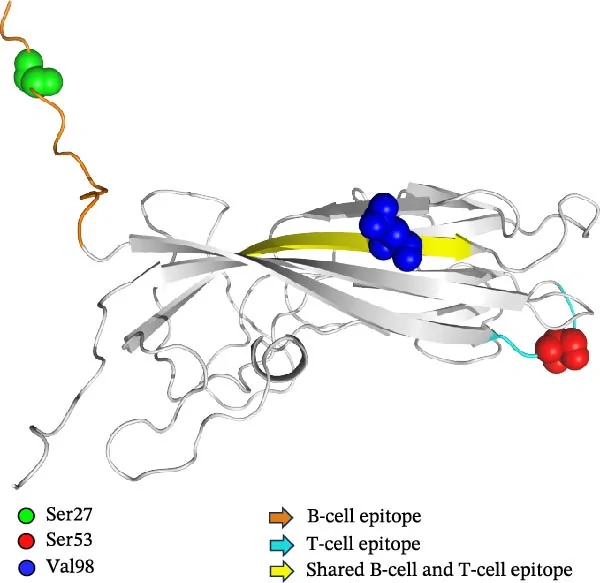
Structural mapping of predicted B‐cell and T‐cell epitopes and candidate positively selected sites on the PCV4 Cap protein monomer.

## 4. Discussion

In this study, we developed a *Rep*‐targeted SYBR Green I real‐time quantitative PCR assay that showed strong linearity (*R* ^2^ = 0.9991), an amplification efficiency of 102.3%, and an estimated LOD95% of 8.22 copies/µL. Compared with the probe‐based TaqMan format, the SYBR Green I assay requires only a primer pair without a sequence‐specific labeled hydrolysis probe, thereby reducing the number of assay‐specific oligonucleotides and potentially facilitating implementation Direct numerical comparison of LOD values among published PCV4 assays is also limited by differences in standard materials, template inputs, replicate designs, positivity criteria, and LOD definitions. Because SYBR Green I generates fluorescence from any double‐stranded DNA product, nonspecific amplicons and primer dimers can contribute to the amplification signal [30, 31]. Although the single melting peak at 85.5 ± 0.5°C supported product specificity, melt curve analysis cannot completely exclude nonspecific products with similar melting temperatures or low‐level background amplification. Coextracted inhibitors in serum, tissue homogenates, fecal material, and necrotic tissues may also impair amplification and cause false‐negative results at low target concentrations; tissue homogenates were evaluated, but matrix‐associated inhibition was not quantified, and the other matrices were not separately validated. Moreover, the complete agreement observed in the evaluated clinical panel should not be interpreted as evidence of equivalent analytical sensitivity or broader diagnostic accuracy because a matched analytical sensitivity comparison was not performed; further validation using larger numbers of low‐target‐concentration specimens and more diverse clinical matrices is required [32, 33].

The assay was subsequently applied to 1920 passive diagnostic submissions received from 29 Chinese provinces between 2022 and 2025, of which 96 (5.00%) tested positive for PCV4. Published positivity estimates vary considerably: PCV4 was detected in 12.8% of clinical samples from Hunan [7], ~17% of samples collected from deceased pigs in Xinjiang [34], and 2.2% of testicular processing fluid samples from suckling pigs in a cross‐sectional survey [35]. These differences illustrate how sampling design, geographic coverage, study population, clinical status, production stage, specimen type, and detection method can influence observed positivity, limiting direct comparison among studies. The same concern applies to the increase observed from 2022 to 2025 in our diagnostic submission dataset because annual differences in submission volume, geographic coverage, herd sources, and clinical case composition may have contributed to the temporal pattern. Accordingly, neither the overall positivity of 5.00% nor the increasing annual trend should be interpreted as a nationally representative prevalence estimate or definitive evidence of a population‐level increase; instead, they describe the observed pattern within this diagnostic submission dataset and highlight the need for continued surveillance with more systematic sampling. The distribution patterns of PCVs appear to vary by age and production stage. A recent survey of intensive pig farms revealed that PCV2 and PCV3 detection peaked in fattening pigs and sows, respectively, whereas PCV4 was detected infrequently across all evaluated age groups [35]. Although PCV4 has also been identified in pigs of various ages and in aborted fetuses [11, 36], these observations and our own findings should be interpreted as epidemiological signals warranting further investigation, rather than definitive evidence of reproductive tropism, reproductive failure causation, or vertical transmission.

RDP5 integrates multiple algorithms to identify mosaic or breakpoint‐associated sequence patterns, whereas GARD evaluates whether candidate breakpoints improve the fit of partition‐specific phylogenetic models. Their different assumptions and search strategies may produce discordant results in short, low‐diversity alignments affected by substitution‐rate heterogeneity or convergent substitutions. In this study, the RDP5 signals were weak, method‐dependent, and classified as potentially attributable to nonrecombinant processes, whereas GARD detected no statistically supported breakpoint. Recombination was therefore not confirmed, and no sequences were excluded on the basis of the recombination screening. Future analyses excluding the sequences associated with the RDP5 signals could provide a conservative assessment of the robustness of the molecular clock and phylogeographic estimates.

The *Cap* gene‐based Bayesian analysis of the final 89‐sequence dataset estimated a tMRCA of ~1996.6 and an evolutionary rate of 4.76 × 10^−4^ substitutions/site/year. The estimated rate was lower than rates reported for PCV2 [37] and broadly comparable to PCV3 [38], whereas the tMRCA was more recent than a previous PCV4 estimate of 1952.48 (95% HPD: 1907.77–1984.83) [39]. This difference may reflect variation in the sampling structure and dataset composition. The tMRCA represents the inferred time to the common ancestor of the sampled PCV4 *Cap* sequences and therefore describes the timescale of lineage diversification rather than the exact date of viral emergence. The evolutionary rate estimate provides baseline information for planning molecular surveillance and periodically reassessing diagnostic binding sites and antigenically relevant regions.

Sampling structure also influences phylogeographic and demographic inference. The geographic links reconstructed among sampled provinces and countries should therefore be regarded as model‐inferred associations rather than verified transmission routes. Uneven geographic representation can affect ancestral‐state reconstruction and root‐location inference, making oversampled locations appear disproportionately prominent and undersampled locations appear isolated in reconstructed dispersal networks [40–42]. Temporal clustering may also affect molecular clock calibration, evolutionary‐rate and tMRCA estimates, and their 95% HPD intervals, as well as Bayesian Skyline trajectories. Accordingly, the apparent recent decline in relative effective population size should not be considered definitive evidence of demographic contraction, and the available data cannot reliably establish the geographic origin, transmission direction, or a primary dissemination hub.

The overlap of the three candidate positively selected sites with predicted B‐cell and/or SLA‐I‐binding regions suggests possible immune‐related selection on the PCV4 Cap protein, broadly consistent with observations in PCV2 [43] and PCV3 [44], where selection frequently occurs in surface‐exposed or antigenic regions. However, these in silico associations do not establish effects on antigenicity, immune escape, receptor interaction, or viral fitness.

Future studies should address both the biological significance of these candidate sites and the robustness of the phylogeographic inferences. Naturally occurring single substitutions and their combined haplotypes could be introduced into a PCV4 infectious clone or replicon to determine their specific roles in viral replication and Cap expression. If technically feasible, rescued recombinant viruses could serve as models to assess changes in replication kinetics, cell tropism, and innate immune activation. Concurrently, wild‐type and mutant Cap proteins or virus‐like particles could be analyzed through antibody binding, competitive ELISA, and serum reactivity profiling, alongside neutralization assays utilizing the recombinant viruses or a validated surrogate model. Furthermore, variant‐specific qPCR could quantify wild‐type–mutant competition, while peptide‐stimulated ELISpot or cytokine assays could evaluate T‐cell responses. For phylogeographic inference, balanced sampling could be complemented by livestock movement, animal trade, farm connectivity, transport network, and market chain data, incorporated as explanatory covariates or connectivity matrices in Bayesian models. Potential biases could be further mitigated by accounting for location‐ and year‐specific sampling intensities if the modeling framework permits. Robustness could be assessed through repeated down‐sampling, leave‐one‐location‐out jackknife analyses, spatiotemporally balanced datasets, and comparisons of ancestral‐state assignments, inferred geographic links, and posterior support across sampling schemes. With more comprehensive data, structured coalescent models could further distinguish geographic population structure from lineage movement.

## 5. Conclusion

The *Rep*‐targeted SYBR Green I real‐time quantitative PCR assay developed in this study provides a practical molecular tool for PCV4 detection and surveillance. Among the tested samples, PCV4 positivity was 5.00%. The higher observed positivity in sows and aborted fetuses warrants further investigation into possible vertical transmission. *Cap* gene‐based phylogenetic, molecular clock, phylogeographic, selection pressure, epitope, and structural analyses generated exploratory hypotheses regarding PCV4 evolutionary history, geographic links, and potential immune‐associated variation.

## Author Contributions

Qigai He and Wentao Li conceived the project, supervised the overall study, and critically revised and edited the manuscript. Yi Lu and Qigai He designed the study. Yi Lu, Yanhong Chen, Tong Xu, Yang Zeng, Ling Dong, and Zhijing Mei performed the bench experiments and data analysis. Yi Lu wrote the first draft of the manuscript.

## Funding

This work was supported by the Key Research and Development Program of Jiangxi Province (Grant 20261BCF320004), the National Natural Science Foundation of China (Grant 32302866), and the self‐designed research project for Hainan Institute of Huazhong Agricultural University (Grant 2024HZAUHNZS005).

## Disclosure

All authors have read, reviewed, and approved the final manuscript.

## Ethics Statement

This study did not involve experimental animals or human participants. The clinical samples used in this study were collected for routine diagnostic purposes, and no additional animal experiments were performed specifically for this research. Therefore, ethical approval was not required.

## Conflicts of Interest

The authors declare no conflicts of interest.

## Supporting Information

Additional supporting information can be found online in the Supporting Information section.

## Supporting information


**Supporting Information 1** Construction of the pET28a‐PCV4*-Rep* standard plasmid.

**Supporting Information 2** Table S1: Metadata of PCV4 *Cap* gene sequences used for maximum‐likelihood phylogenetic analysis.

**Supporting Information 3** Table S2: Metadata of PCV4 *Cap* gene sequences used for Bayesian phylodynamic and phylogeographic analyses.

**Supporting Information 4** Table S3: Posterior parameter estimates and convergence diagnostics from two independent strict‐clock Bayesian Skyline analyses of the PCV4 *Cap* gene dataset: (A) run‐specific estimates and diagnostics for run 1 and run 2; and (B) posterior estimates and diagnostics obtained after combining the post‐burn‐in outputs from both runs.

**Supporting Information 5** Table S4: Predicted B‐cell and T‐cell epitopes of the PCV4 Cap protein and their overlap with candidate positively selected sites.

## Data Availability

All newly generated PCV4 *Cap* (ORF2) gene sequences have been submitted to the GenBank. The accession numbers will be provided upon release/acceptance.
