# Supplementary material for: Development and Application of a Rep‐Targeted SYBR Green I qPCR Assay for Porcine Circovirus 4: Etiological Investigation in China (2022–2025) and Molecular Evolutionary Analysis
Source: Transbound Emerg Dis. 2026 Nov 6;2026:4853360. doi: 10.1155/tbed/4853360 (PMC13743856; doi:10.1155/tbed/4853360)
Supplement: Supplementary file 1 [file TBED-2026-4853360-s001.docx]

1. Primers used for construction of the pET28a-PCV4-Rep standard plasmid.
Table S1-1. Primers used for construction of the pET28a-PCV4-Rep standard plasmid.

| Primer | Sequence (5′–3′) | Restriction enzyme recognition site | Expected amplicon size (bp) |
| --- | --- | --- | --- |
| PCV4-*Rep*-Cloning-F | GTGGATCCGAATTCGAGCTCAGTACGCTGTTGTCGG | SacI (GAGCTC) | 169bp |
| PCV4-*Rep*-Cloning-R | CGGCCGCAAGCTTGTCGACGGCTCAATATGGCTCCG | SalI (GTCGAC) |  |
| pET28a-linear-F | TATTGAGCCGTCGACAAGCTTGCGG | SalI (GTCGAC) | 5648bp |
| pET28a-linear-R | AACAGCGTACTGAGCTCGAATTCGGATCCA | SacI (GAGCTC) |  |

2. The amplified vector and *Rep* fragment were subsequently digested with SacI and SalI and ligated using T4 DNA ligase. The ligation product was transformed into chemically competent *Escherichia coli* DH5α cells (TOLOBIO, Beijing, China; Cat. No. CC96102-02). Individual colonies were initially screened by colony PCR. Positive clones were subsequently cultured, after which recombinant plasmid DNA was extracted and subjected to Sanger sequencing by Beijing Tsingke Biotech Co. (Beijing, China) for sequence verification.

Figure S1. PCR verification of the pET28a-PCV4-Rep standard plasmid. M, DL2000 DNA marker; lane 1, PCR product amplified from the candidate recombinant plasmid using the PCV4-Rep-F/R primer pair, with an expected size of 129 bp; lane 2, no-template control.

3.Sanger sequencing verification
Sanger sequencing generated a 997-base read that completely covered the 129-bp PCV4 *Rep* insert. Within the insert, the mean Phred quality score was 47.1. All 129 bases had quality scores ≥Q20, and 127/129 bases (98.45%) had quality scores ≥Q30. Alignment with the intended PCV4 *Rep* target confirmed 100% nucleotide identity across the complete insert, with no substitutions, insertions, or deletions.

Figure S2. Sanger sequencing verification of the pET28a-PCV4-*Rep* standard plasmid.
